# Quercetin ameliorates obesity and inflammation via microbial metabolite indole-3-propionic acid in high fat diet-induced obese mice

**DOI:** 10.3389/fnut.2025.1574792

**Published:** 2025-04-16

**Authors:** Jiaxin Lu, Yanting Huang, Yujing Zhang, Jiayu Xie, Qingjun Guo, Huifan Yang, Yunyan Yang, Jing Chen, Lijie Su

**Affiliations:** ^1^Department of Nutrition and Food Hygiene, School of Public Health, Guangzhou Medical University, Guangzhou, Guangdong, China; ^2^Sino-French Hoffmann Institute, School of Basic Medical Science, Guangzhou Medical University, Guangzhou, Guangdong, China

**Keywords:** quercetin, indole-3-propionic acid, tryptophan metabolite, gut microbiota, obesity

## Abstract

**Background:**

Obesity is a chronic metabolic disease, mainly caused by excessive/abnormal fat accumulation, as well as accompanied by endotoxemia and chronic inflammation. Quercetin, a natural flavonoid, may alleviate obesity by regulating gut microbiota and metabolites, but its exact mechanism for improving obesity is unknown.

**Objectives:**

The aim of this study was to investigate the effects of quercetin on high-fat diet (HFD)-induced obesity in mice. In particular, we focused on the regulatory effects of quercetin on gut microbiota and the tryptophan metabolite indole-3-propionic acid (IPA).

**Methods:**

The C57BL/6J mice were subjected to a 20-week HFD feeding regimen with concurrent daily oral administration of quercetin or IPA. The body weight, fat accumulation, gut barrier function, and chronic inflammation were determined. Gut microbiota composition was analyzed by 16S rRNA sequencing and IPA levels were measured in serum and feces. *In vitro* experiments, Caco-2 cells were used to evaluate the effects of IPA and fecal dilutions from quercetin-treated mice on tight junction protein expression and aryl hydrocarbon receptor (AhR) activation.

**Results:**

Our results revealed that quercetin supplementation significantly mitigated obesity and chronic inflammation, and improved the disrupted gut barrier function through the actvation of AhR/interleukin 22 (IL-22) pathway. 16S rRNA sequencing revealed that quercetin treatment increased the abundance of *Lactobacillus*. Quercetin intervention increased the levels of IPA in the serum and feces of mice. IPA supplementation alleviated obesity and chronic inflammation, and enhanced intestinal barrier function through AhR activation. The findings were further corroborated by Caco-2 cell experiment, which indicated that the modulation of the dysregulated gut microbiota to change microbial metabolite IPA coordinated the improvement effect of quercetin on gut barrier disruption.

**Conclusion:**

Quercetin supplementation alleviates obesity by restoring high-fat diet induced gut microbiota disorder, which elevates IPA level to activate AhR/IL-22 pathway, thereby enhancing intestinal barrier integrity and suppressing chronic inflammation.

## 1 Introduction

Obesity is a chronic multisystem disease, and the increasing prevalence of obesity represents a significant healthcare challenge in both developed and developing countries (1). The contributing factors to obesity include genetics, diet, unhealthy lifestyle, inflammation, and gut microbiota. Among these, disruption of the gut microbiota is a key factor in the development of obesity. Cani et al. (2) demonstrated that the high-fat diet causes a disturbance in the gut microbiota, resulting in damage to the intestinal barrier, and increased plasma levels of lipopolysaccharides (LPS), also known as metabolic endotoxemia. A chronic low-grade inflammation caused by endotoxemia is responsible for the development of obesity. Therefore, improving the gut barrier damage to alleviate chronic inflammation may be an effective therapeutic strategy for obesity treatment.

Tryptophan is an essential aromatic amino acid that plays a key role in intestinal homeostasis (3). Tryptophan metabolism follows three major pathways involving serotonin, kynurenine, and gut microbiota (4). The microflora of *Lactobacillus, Bifidobacterium*, and *Peptostreptococcus* can metabolize tryptophan to produce a variety of indole derivatives (5), which are ligands for the aryl hydrocarbon receptor (AhR) and stimulate the expression of interleukin 22 (IL-22), thereby regulating the intestinal mucosal homeostasis (6). Indole-3-propionic acid (IPA), an indole derivative produced solely by gut bacteria, has been shown to alleviate hyperlipidemia and non-alcoholic fatty liver disease (7, 8). Population studies have shown that serum IPA levels are significantly lower in obese people (9), raising the possibility that IPA may be a biomarker for metabolic diseases. A recent study has demonstrated that IPA enhances intestinal barrier function and may mitigate the inflammatory damage caused by LPS in human colonic epithelial cells (10). Therefore, IPA may serve as a novel therapeutic agent for obesity treatment by restoring microbial homeostasis and modulating dysregulated microbial metabolites.

Quercetin is a natural flavonol with a variety of biological activities, including antioxidants, lipid-lowering, tumor inhibition, and immunomodulatory function (11). In recent years, quercetin has attracted much attention for the remarkable beneficial effects in regulating lipid metabolism, improving insulin resistance, and alleviating obesity-related inflammation and metabolic disorders (12–14). While the anti-obesity effects of quercetin have been linked to the restoration of the balance of the gut microbiota (15), the precise mechanisms remain unclear. Emerging evidence suggests that flavonoids, including quercetin, protect the intestinal barrier by modulating gut microbiota metabolites (16). For example, dihydromyricetin restores intestinal barrier function via bile acids and also improves insulin resistance by regulating gut microbiota and metabolites, particularly chenodeoxycholic acid levels (17, 18). Notably, the microbial metabolite IPA, derived from tryptophan, not only reduces the symptoms of obesity, but is also a biomarker for obesity (19, 20). IPA production by *Lactobacillus* also activates the AhR pathway, which may restore intestinal barrier integrity (21). Supplementation with mulberry-derived 1-deoxynojirimycin has been shown to modulate gut microbiota and increase IPA levels, exerting a lipid-lowering effect (7). Nonetheless, further research is needed to elucidate the specific mechanisms underlying the role of IPA in ameliorating obesity. Based on these findings, we hypothesize that quercetin ameliorates high-fat diet-induced obesity by modulating gut microbiota composition (such as *Lactobacillus*) and enhancing IPA production.

In this study, we examined the impact of quercetin on HFD-induced obese mice. Our findings demonstrated that quercetin effectively mitigated obesity and chronic inflammation, while also regulating microbial composition to promote the production of IPA. Furthermore, IPA supplementation in obese mice showed that it could effectively alleviate obesity and chronic inflammation, and the improvement effect may be related to the activation of AhR/IL-22 pathway to enhance intestinal barrier function. Overall, our findings will provide new insights into the underlying mechanisms by which quercetin alleviated obesity through the modulation of gut microbiota and microbial tryptophan metabolites.

## 2 Materials and methods

### 2.1 Animal and treatment

The C57BL/6J mice were purchased from the Guangdong Province Medical Experimental Animal Center. The experiment was approved by the Ethics Committee of Experimental Animals of Guangzhou Medical University (permit No. GY2022-084). The mice (~22g) are bred in the SPF-level animal laboratory of the experimental animal center of Guangzhou Medical University after 2 weeks of adaptive feeding. The mice in this experiment were provided with ad libitum access to food and maintained under controlled environmental conditions: ambient temperature of 22 ± 1°C, relative humidity of 50–60%, and a 12 h light/dark cycle.

Firstly, in order to verify the effect of quercetin on obesity, we performed animal experiments. Mice were adaptively fed for 7 days and randomly assigned to one of three groups (*n* = 5 in each group) based on body weight: a low-fat diet group (LFD, D12450H, 10% kcal fat), a high-fat diet group (HFD, D12451, 45% kcal fat), a high-fat diet supplemented with quercetin group (HFD+Q, 97% purity, Macklin, 50 mg/kg/day). The selection of quercetin concentration was based on our previously published studies (22). During the experiment, body weight was recorded weekly, and the food intake was recorded every 2 days. After 20 weeks of gavage, the mice were anesthetized by intraperitoneal injection of 50 mg/kg of sodium pentobarbital. Blood samples were collected through retro-orbital bleeding and allowed to clot at room temperature for 30 min. Serum was separated by centrifugation at 3,000 × g for 15 min at 4°C and stored at −80°C. Subsequently, tissues including liver, colon, feces, and peritesticular fat were collected from each group in turn (*n* = 5). The tissues were weighed and dated immediately after dissection.

Secondly, the effect of IPA on obesity was verified by IPA supplementation experiment. The mice were divided into three groups (*n* = 5 in each group) based on body weight: a low-fat diet group (LFD, D12450H, 10% kcal fat), a high-fat diet group (HFD, D12451, 45% kcal fat), and a high-fat diet supplemented with IPA (HFD + IPA, 98% purity Macklin, 50 mg/kg/day). The selection of IPA concentration was based on previously published papers (23). The experimental conditions and methods were in accord with the previous quercetin intervention experiments.

### 2.2 Histopathological examination

Liver tissues were rinsed with saline, fixed in 4% paraformaldehyde, and processed for lipid deposition analysis. Sections were stained with 0.5% Oil Red O (15 min), differentiated in 60% isopropanol, and counterstained with hematoxylin. For quantification, frozen liver tissues embedded in OCT compound were stained with Oil Red O, and lipid-positive areas were measured using ImageJ. Peritesticular adipose tissues were fixed in 10% formalin overnight, dehydrated through graded ethanol, paraffin-embedded, and sectioned (5 μm). Sections were stained with hematoxylin and eosin (H&E). Adipocyte diameters were quantified by analyzing five random fields per section using ImageJ.

### 2.3 Biochemical analysis

A 96-well plate was divided into three groups (three wells per sample): blank control, standard (for calibration curve using serial dilutions), and test samples. Antibodies were added and incubated according to the manufacturer's protocol. After washing, samples were added to the substrate and incubated at 37°C (protected from light) for 10 min. Measurement with an enzyme marker according to the wavelength of the different substances to be measured. Target concentrations were calculated using the standard curve (OD vs. concentration). Serum total cholesterol (TC), triglyceride (TG), and insulin levels were analyzed with commercial kits (Nanjing Jiancheng Bioengineering). LPS, tumor necrosis factor-alpha (TNF-α), interleukin-1 beta (IL-1β), and IL-22 were quantified via enzyme-Linked immunosorbent assay (ELISA: Meimian, Zci Bio kits) following manufacturer instructions.

### 2.4 RT-qPCR

Total RNA was isolated from liver, colon, and peritesticular fat tissues (*n* = 3 biological replicates per group) using TRIzol™ reagent (Biosharp, China) following the manufacturer's protocol. Briefly, tissues were homogenized in 1 mL TRIzol using a bead-based homogenizer (Precellys 24, Bertin Technologies), followed by phase separation with chloroform. RNA was precipitated with isopropanol, washed with 75% ethanol, and dissolved in RNase-free water. RNA concentration and purity were assessed spectrophotometrically (A260/A280 ratio ≥ 1.8; NanoDrop 2000, Thermo Fisher). qPCR was performed using SYBR green PCR kit. qPCR was performed using SYBR green PCR kit. The primer sequences were listed in Table 1. The relative gene expressions were quantified using 2^−ΔΔCT^ method.

**Table 1:** The primer sequence of genes for qPCR.

**Gene**	**Primer sequence**	
*Tnf-*α	Forward	CAGGCGGTGCCTATGTCTC
	Reverse	CGATCACCCCGAAGTTCAGTAG
*Il-6*	Forward	TAGTCCTTCCTACCCCAATTTCC
	Reverse	TTGGTCCTTAGCCACTCCTTC
*Il-1*β	Forward	TTCAGGCAGGCAGTATCACTC
	Reverse	GAAGGTCCACGGGAAAGACAC
*Mucin-2*	Forward	ATGCCCACCTCCTCAAAGAC
	Reverse	GTAGTTTCCGTTGGAACAGTGAA
*Occlaudin*	Forward	TTGAAAGTCCACCTCCTTACAGA
	Reverse	CCGGATAAAAAGAGTACGCTGG
*Zo-1*	Forward	GCTTTAGCGAACAGAAGGAGC
	Reverse	TTCATTTTTCCGAGACTTCACCA
*GAPDH*	Forward	TGTTTCCTCGTCCCGTAG
	Reverse	CAATCTCCACTTTGCCACT
β*-Actin*	Forward	GGCTGTATTCCCCTCCATCG
	Reverse	CCAGTTGGTAACAATGCCATGT

### 2.5 Western blot analysis

Colon tissues (*n* = 3 biological replicates per group) were homogenized in ice-cold PBS with steel beads and lysis buffer. Lysates were centrifuged (12,000 × g, 15 min, 4°C), and supernatants were quantified using a BCA assay. Proteins were normalized, denatured in Laemmli buffer (95°C, 5 min), and stored at −20°C. Proteins were separated on 7.5% SDS-PAGE gels and transferred to methanol-activated PVDF membranes (0.45 μm, 240 mA, 40 min). Membranes were blocked with 5% non-fat milk in TBST (2 h, room temperature), incubated with primary antibodies overnight at 4°C, then with HRP-conjugated secondary antibodies (1:5,000, 1 h, room temperature). Antibodies and Detection: AhR (1:2,000; Goat #AF6278, Affinity Biosciences); β-actin (1:5,000; Mouse #AB6276, Abcam). HRP-conjugated secondary antibodies: Goat Anti-Rabbit IgG (1:5,000; #S0001); Goat Anti-Mouse IgG (1:5,000; #S0002). Protein bands were visualized using Western Lightning Plus-ECL reagent (Bio-Rad) and quantified by densitometry with ImageJ.

### 2.6 Bioinformatics analysis

Fecal samples (~10 mg per group; *n* = 5 biological replicates) were randomly selected for 16S rRNA sequencing. Microbial DNA was extracted using the HiPure Stool DNA Kits (Magen, Guangzhou, China) according to the manufacturer's protocols. The V3 and V4 region of the 16S rRNA gene (primers 341F: 5′-CCTACGGGNGGCWGCAG-3′ and 806R: 5′-GGACTACHVGGGTATCTAAT-3′) were amplified by PCR and sequenced on Illumina Hiseq 2,500 (Genedenovo Biotechnology Co., Ltd, Guangzhou, China). To get high-quality clean reads, raw reads were further filtered using FASTP, and paired-end clean reads were merged as raw tags using FLSAH. The effective tags were clustered into operational taxonomic units (OTUs) using the UPARSE pipeline. The tag sequence with the highest abundance was selected as a representative sequence within each cluster. Venn diagrams (R software) identified shared/unique OTUs. Beta diversity was analyzed via non-metric multidimensional scaling (NMDS; Bray-Curtis). Taxonomic differences were assessed using Kruskal-Wallis test with FDR correction (*p* < 0.05). Biomarker features in each group were screened by Metastat and LEFSE software. A statistically significant difference was defined as *p* < 0.05 and an LDA score of 3 or above.

### 2.7 IPA measurement

The blood samples were obtained from mice via the inner canthus following a 6 h fasting period. Then, the serum was subsequently isolated and stored at −80°C until further analysis. On a daily basis, two to three fecal pellets from each group of mice were collected and combined in sterile tubes. 10 mg of feces were dissolved in 1 ml of sterile saline, which were subjected to centrifugation at 8,000 × g at 4°C for 5 min. The fecal dilutions were collected and stored at −20°C. At last, the concentrations of IPA in the serum and fecal dilutions were examined by ELISA kits (Zci Bio, China).

### 2.8 Cell culture and treatments

Caco-2 cells were obtained from the Chinese Academy of Sciences Cell Bank (Shanghai, China). Caco-2 cells were cultured in DMEM containing 10% fetal bovine serum and 1% non-essential amino acid solution (Penicillin-Streptomycin, Gibco). Cells were seeded into 12-well transwell^®^ collagen-coated inserts at a density of 1 × 10^5^ cells/well and cultured in DMEM for 21 days for cell monolayer development. The transepithelial electric resistance (TEER) of the treated cell monolayers was determined at 37°C by the epithelial voltammeter (Millicell^®^ ERS-2, USA). TEER value of 400–600 Ω·cm^2^ indicated the formation of tight junctions between cells, then cells were treated with LPS, LPS+IPA (0.1, 0.5, 1 mM), and fecal dilutions from quercetin-treated mice (abbreviated as LPS+QF group) for 24 h, respectively. After the FD40 concentration was measured at 480 nm excitation and 520 nm emission wavelengths, the cell barrier permeability was compared using the standard curve.

Cells were then rinsed, scraped and collected for Western blotting analysis. The following antibodies were used in the Caco-1 cells: AhR (1:5,000, Mouse#67785-1-Ig; Proteintech), GAPDH (1:5,000, Goat#AF7021; Affinity Biosciences), Zo-1 (1:2,000, Goat# #AF5145; Affinity Biosciences), Claudin1 (1:1,000, Goat#AF0127; Affinity Biosciences).

### 2.9 Statistical analysis

Data are expressed as mean ± SD from three independent experiments. Normality was confirmed using the Shapiro-Wilk test. Statistical analysis was performed with SPSS 26.0 and GraphPad Prism 9. One-way analysis of variance (ANOVA) followed by Dunnett's *post hoc* test (for multiple comparisons) was applied to compare differences among groups, with *p* < 0.05 considered statistically significant.

## 3 Results

### 3.1 Quercetin ameliorated HFD-induced obesity in mice

**Figure 1:**
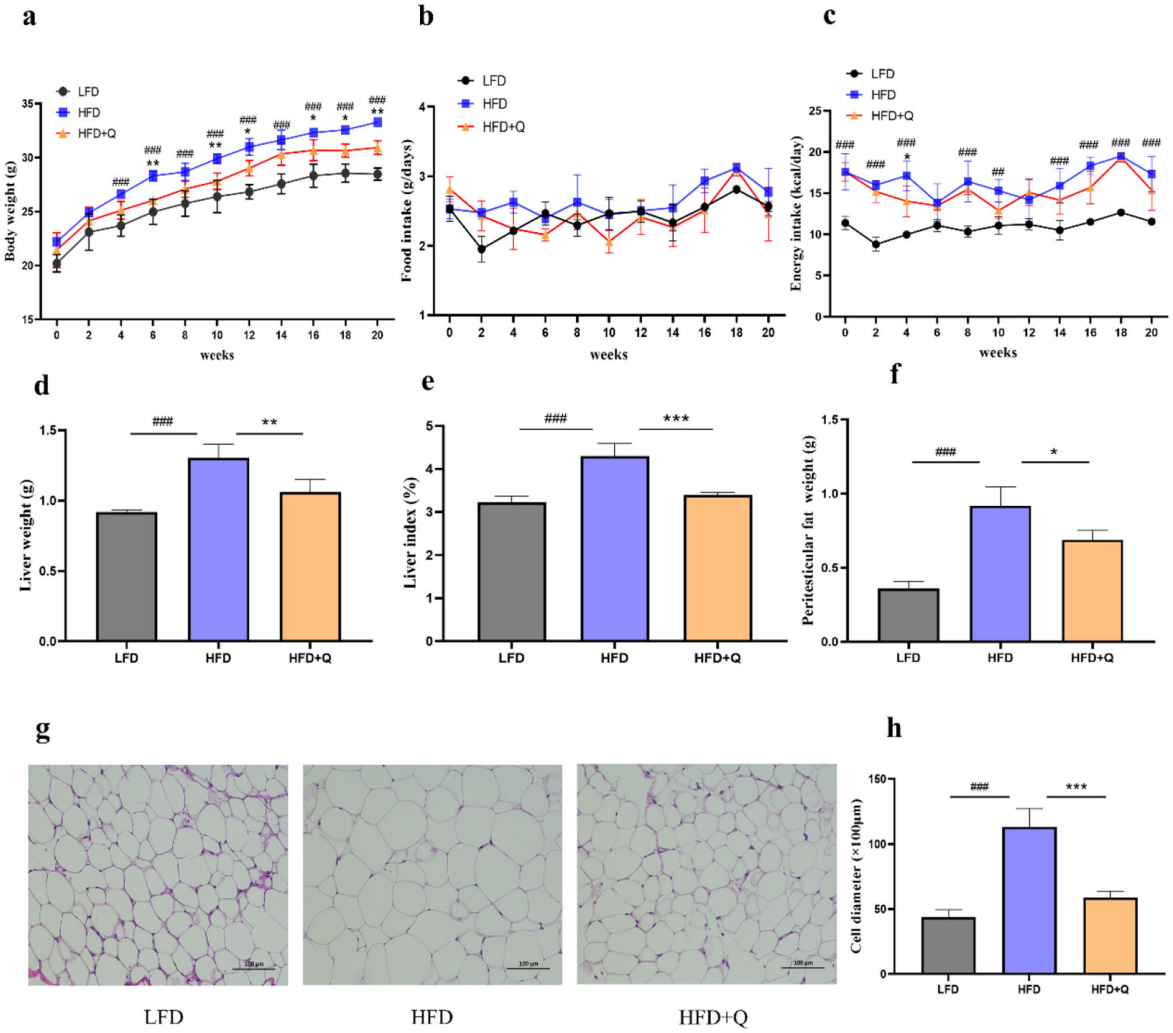
Administration of quercetin improved obesity characteristics in mice fed with high-fat diet. **(a)** The body weight. **(b)** The food intake. **(c)** The energy intake. **(d)** The liver weight. **(e)** The Liver index. **(f)** The weight of peritesticular adipose tissue. **(g)** H&E staining of peritesticular adipose tissue sections (Scale bar: 100 μm). **(h)** The adipocyte diameter size. Data were mean ± SEM, *n* = 5. Pound indicated significant differences between LFD vs. HFD (^##^*p* < 0.01; ^###^*p* < 0.001). Asterisk indicated significant differences between HFD vs. HFD+Q (*p < 0.05; **p < 0.01; ***p < 0.001).

The body weight of mice in the HFD group was significantly higher than that in the LFD group, the administration of quercetin significantly alleviated this trend (Figure 1a). No significant differences in food intake of mice among the three groups were observed (Figure 1b). However, the energy intake of mice in the HFD group was significantly higher than that in the LFD group, and there was no significant difference in energy intake between the HFD group and the HFD+Q group (Figure 1c). Quercetin was also effective in reducing the weight of the liver and the liver index in HFD-fed mice (Figures 1d, e). Peritesticular fat weight was significantly increased in the HFD group as compared with that in the LFD group, while quercetin intervention inhibited the increase of peritesticular fat weight (Figure 1f). Histological examination showed that the diameter of adipocytes in the HFD group was excessively increased compared with that in the LFD group. However, supplementation with quercetin prominently prevented the excessive enlargement of adipocytes (Figures 1g, h).

### 3.2 Quercetin effectively improved the glucose and lipid metabolism disorders in obese mice

**Figure 2:**
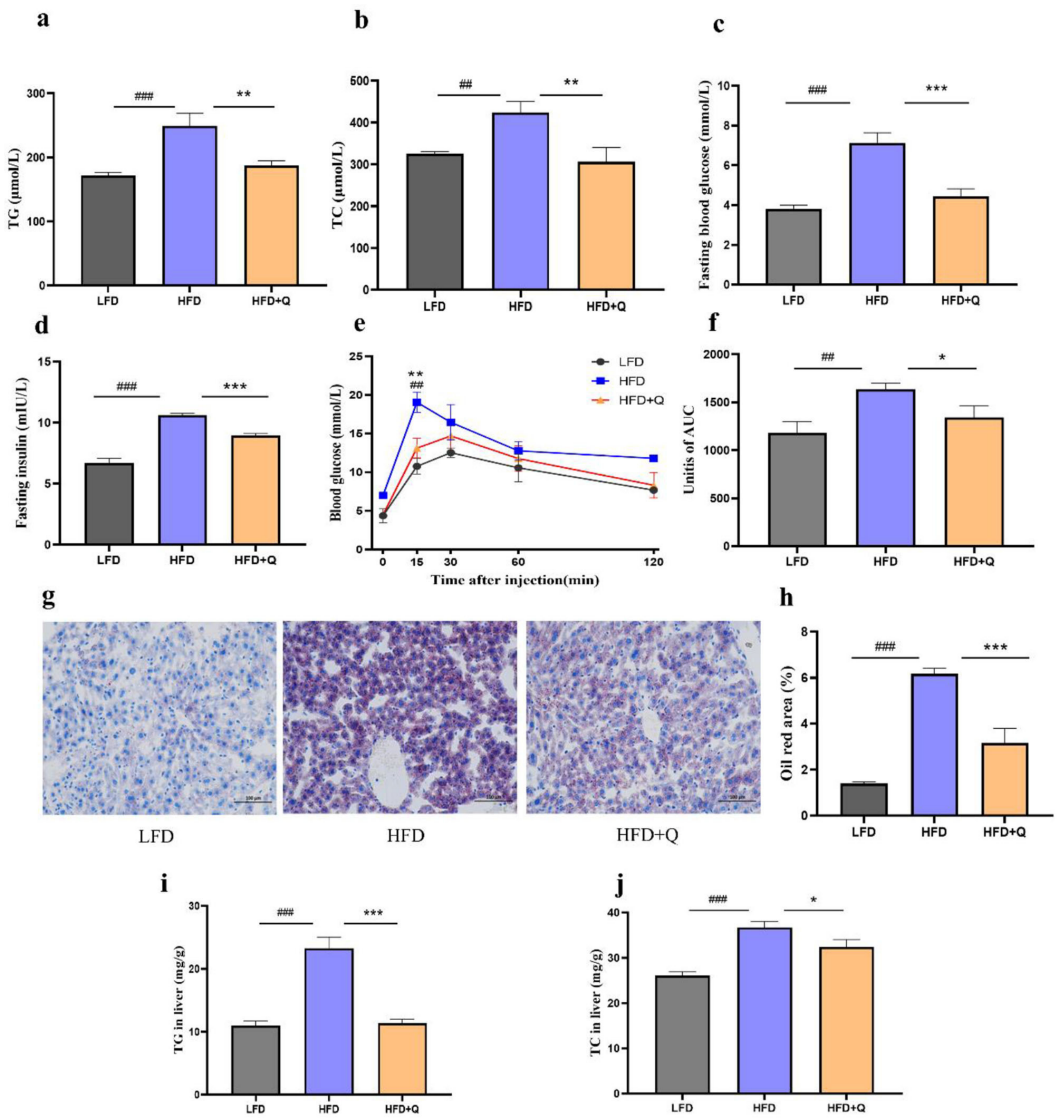
Quercetin improved the metabolic parameters of mice fed with high-fat diet. **(a)** TG level in serum of mice. **(b)** TC level in serum of mice. **(c, d)** The blood glucose and insulin levels after fasting for 8 h. **(e)** Time-dependent profiles of blood glucose levels in OGTT. **(f)** The mean AUC from OGTT. **(g)** Oil red O staining of lipid droplets in liver (Scale bar: 100 μm). **(h)** Oil Red O percent stained area. **(i)** TG level in liver of mice. **(j)** TC level in liver of mice. Data were mean ± SEM, *n* = 5. Pound indicated significant differences between LFD vs. HFD (^##^*p* < 0.01; ^###^*p* < 0.001). Asterisk indicated significant differences between HFD vs. HFD+Q (*p < 0.05; **p < 0.01; ***p < 0.001).

The serum levels of TG and TC were significantly increased in the HFD group compared to those in the LFD group, which were reversed upon quercetin treatment (Figures 2a, b). Fasting blood glucose and insulin levels were significantly higher in HFD-fed mice compared with those in LFD-fed mice, and quercetin treatment effectively attenuated HFD-induced elevations (Figures 2c, d). During OGTT, the HFD-fed mice had significantly higher blood glucose levels at all time points compared to the LFD-fed mice (Figure 2e). Quercetin treatment decreased blood glucose levels from 15 to 120 min, especially in 15 min. Consistently, the mice of the HFD group had the highest AUC value, while quercetin supplementation significantly reduced the AUC value (Figure 2f). Moreover, HFD caused more lipid deposition in liver, and quercetin intervention significantly reduced the lipid droplet area (Figures 2g, h). The levels of TG and TC in the liver of HFD-fed mice were significantly increased, but this phenomenon was reversed after quercetin treatment (Figures 2i, j).

### 3.3 Quercetin alleviated chronic inflammation and impairment of intestinal barrier function in obese mice

**Figure 3:**
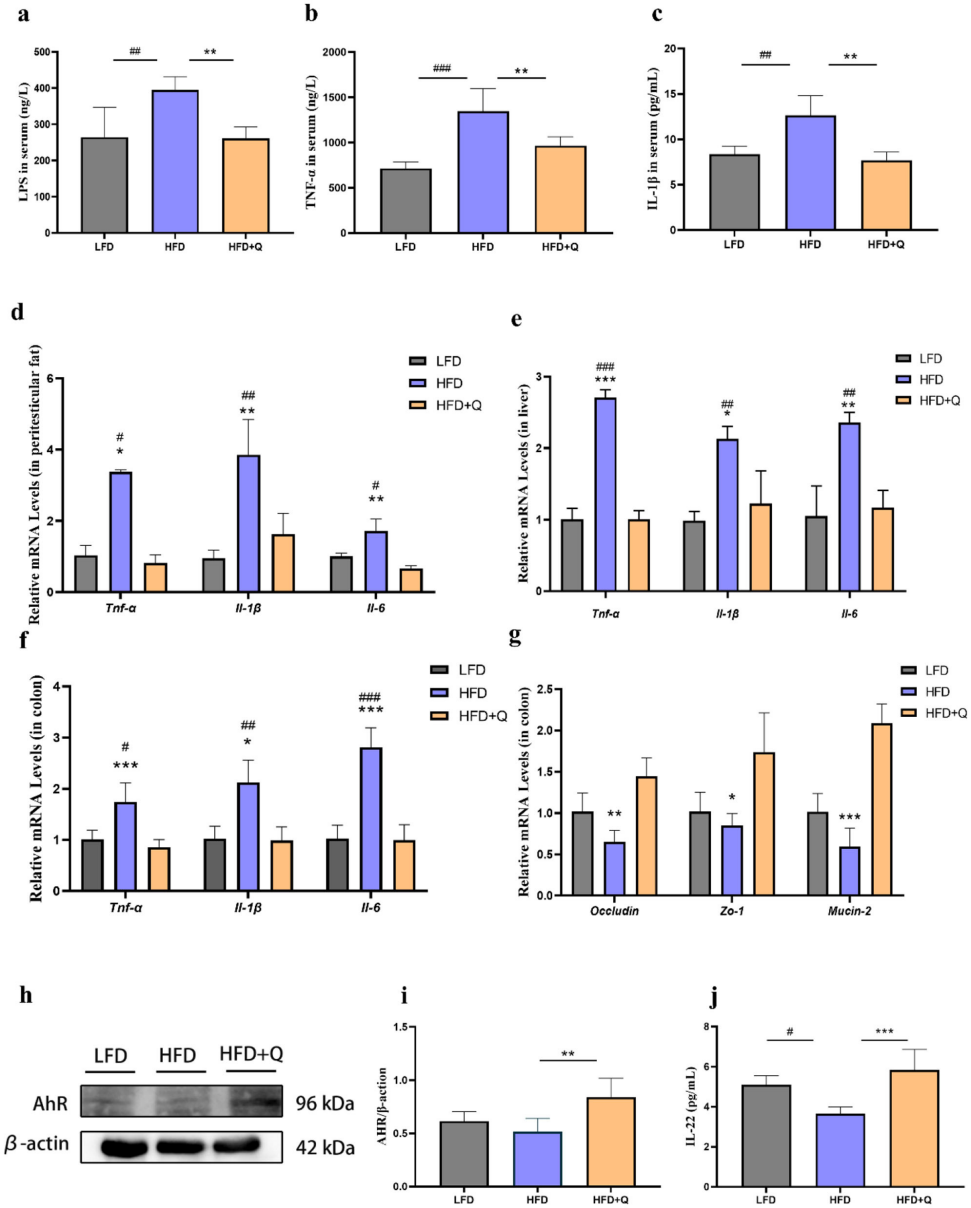
Quercetin ameliorated intestinal barrier damage and endotoxemia in mice fed with high-fat diet. **(a)** LPS level in serum of mice. **(b, c)** Levels of TNF-α and IL-1β in serum of mice. **(d)** Expression levels of genes associated with inflammation in peritesticular adipose tissue. **(e)** Expression levels of genes associated with inflammation in liver. **(f)** Expression levels of genes associated with inflammation in colon. **(g)** Expression levels of intestinal barrier genes in colon. **(h, i)** AhR protein expression in colon of mice. **(j)** IL-22 level in colon of mice. Data were mean ± SEM, *n* = 5. Pound indicated significant differences between LFD vs. HFD (^#^*p* < 0.05; ^##^*p* < 0.01; ^###^*p* < 0.001). Asterisk indicated significant differences between HFD vs. HFD+Q (*p < 0.05; **p < 0.01; ***p < 0.001).

Compared to the LFD group, the mice in the HFD group had significantly higher serum levels of LPS, TNF-α, and IL-1β. This HFD-induced elevation was ameliorated by quercetin treatment (Figures 3a–c). Consistently, the mRNA expressions of *Tnf-*α, *Il-6*, and *Il-1*β were significantly increased in adipose tissue, liver, and colon of mice fed with HFD. Nevertheless, quercetin treatment reduced the mRNA expressions of these cytokines (Figures 3d–f). The mRNA expressions of *Occludin, Zo-1*, and *Mucin-2* associated with the intestinal barrier function were significantly reduced in mice of HFD group, and these reductions were reversed after quercetin treatment (Figure 3g). Then, the role of quercetin in the activation of AhR was investigated. Lower AhR activity was observed in the colon of HFD-fed mice, which was reversed upon quercetin treatment (Figures 3h, i). Moreover, given the critical role of IL-22 in promoting intestinal barrier integrity, we hypothesized that quercetin supplementation could promote the level of IL-22 (Figure 3j). The result showed that quercetin supplementation could significantly increase the expression of IL-22 in colon.

### 3.4 Quercetin ameliorated gut microbiota disturbance in HFD-induced obese mice

**Figure 4:**
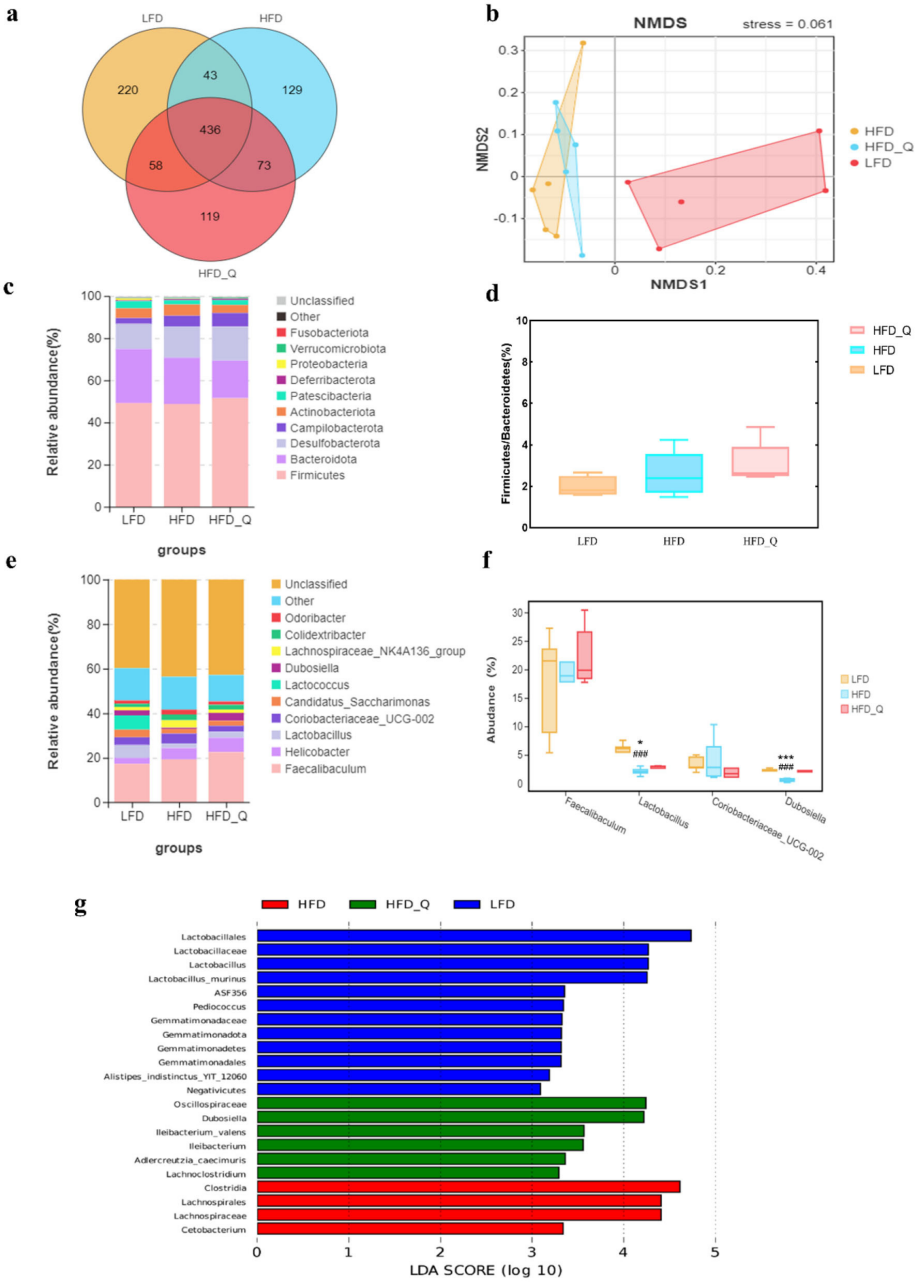
The effects of quercetin on gut microbiota in high-fat diet mice. **(a)** Venn diagram. **(b)** NMDS analysis. **(c)** The result of phylum level analysis. **(d)** The ratio of *Firmicutes* to *Bacteroidetes*. **(e)** The result of genus level analysis. **(f)** Relative abundance of the representative bacterial genus. **(g)** The result of LEFSE analysis. Data were mean ± SEM, *n* = 5. Pound indicated significant differences between LFD vs. HFD (^###^*p* < 0.001). Asterisk indicated significant differences between HFD vs. HFD+Q (*p < 0.05; ***p < 0.001).

The Venn diagram analysis showed that 436 OTUs were shared among the three groups, and 119 OTUs were unique in the HFD+Q group (Figure 4a). Furthermore, beta diversity analysis results indicated that the gut microbiota composition differed significantly between the HFD group and the LFD group, and a slight difference was observed between the HFD+Q group and the HFD group (Figure 4b). At the phylum level, mice fed with HFD exhibited a relatively higher abundance of *Firmicutes* and a lower abundance of *Bacteroidetes* (Figure 4c). Quercetin treatment increased the *Firmicutes*/*Bacteroidetes* ratio and decreased the abundance of *Bacteroidetes* (Figure 4d). At the genus level, the HFD group showed an increase in the abundance of *Lachnospiraceae_NK4A136_group* and *Coriobacteriaceae-UCG-002* (Figure 4e). It is worth noting that quercetin supplementation increased the abundance of *Faecalibacterium, Lactobacillus*, and *Dubosiella* (Figure 4f). LEFSE analysis revealed a significant increase in *Lactobacillales* in the LFD group, while *Clostridia* and *Lachnospirares* significantly increased in mice fed with HFD. Quercetin treatment significantly elevated the levels of *Dubosiella* and *Lleibacterium* genus (Figure 4g).

### 3.5 Quercetin intervention altered the level of IPA in obese mice

**Figure 5:**
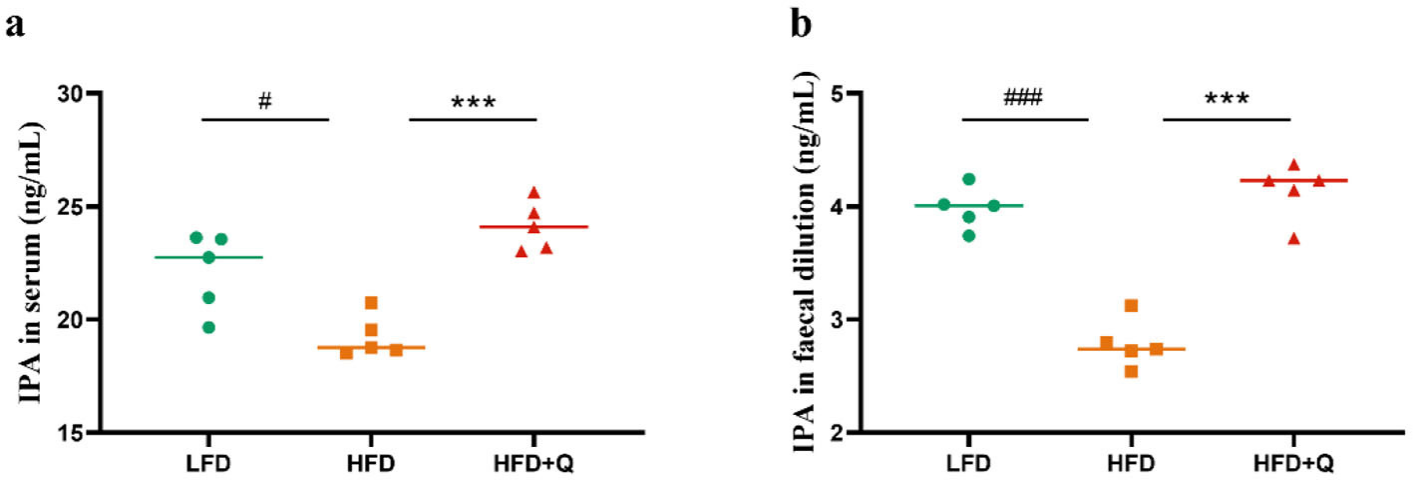
The IPA levels in serum and feces of mice fed with high-fat diet. **(a)** Concentration of IPA in serum. **(b)** Concentration of IPA in feces. Data were mean ± SEM, *n* = 5. Pound indicated significant differences between LFD vs. HFD (^#^*p* < 0.05; ^###^*p* < 0.001). Asterisk indicated significant differences between HFD vs. HFD+Q (***p < 0.001).

Given that quercetin promotes the abundance of *Lactobacillus*, we hypothesized that quercetin could enhance the microbial tryptophan metabolism to regulate the level of IPA. The IPA levels in the serum and feces of mice in HFD group were significantly lower than those in the LFD group (Figures 5a, b), and those reduction were reversed by quercetin treatment.

### 3.6 IPA ameliorated HFD-induced obesity in mice

**Figure 6:**
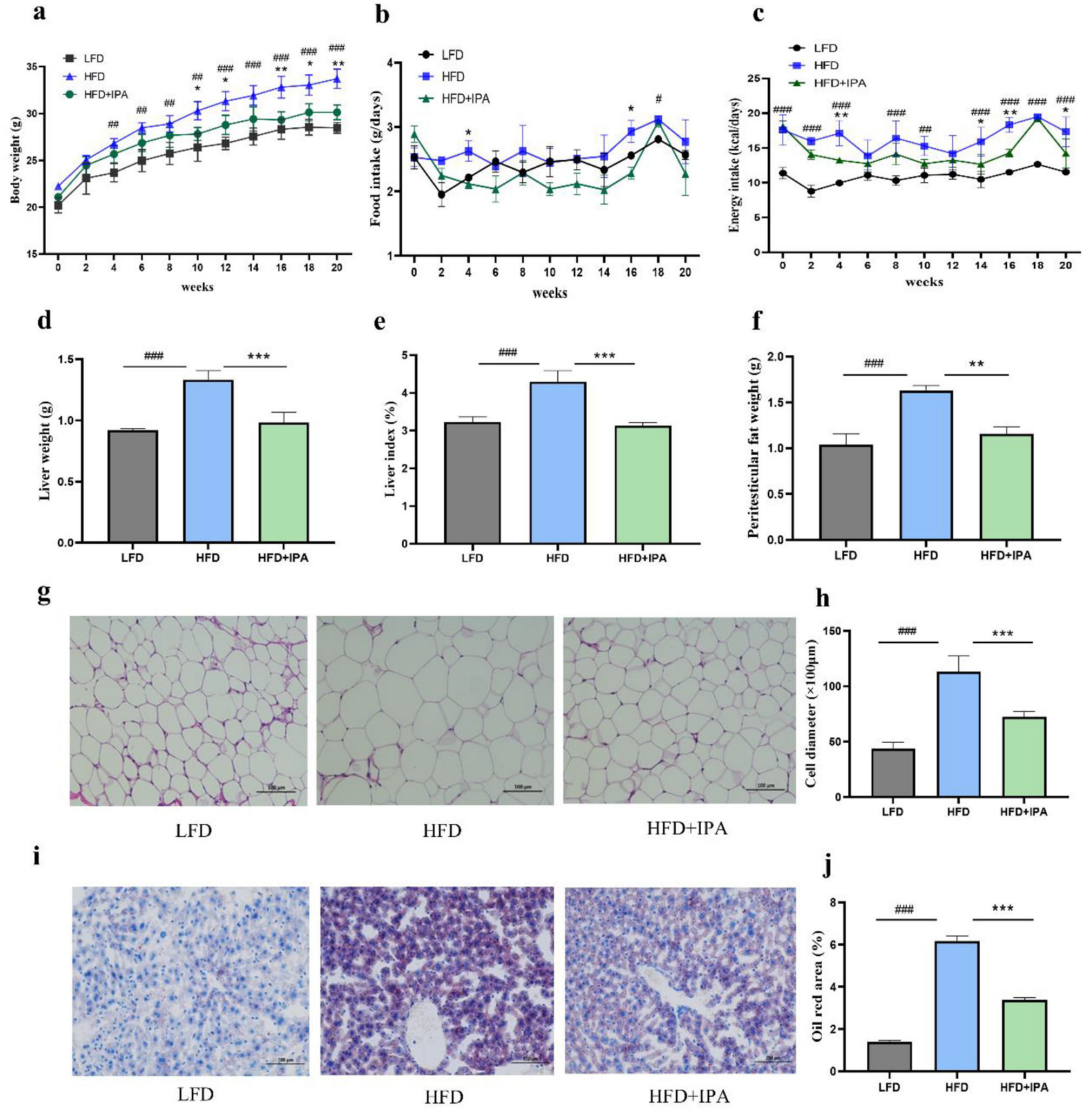
Administration of IPA improved obesity characteristics in mice fed with high-fat diet. **(a)** The body weight. **(b)** The food intake. **(c)** The energy intake. **(d)** The liver weight. **(e)** The liver index. **(f)** The weight of peritesticular adipose tissue. **(g, h)** H&E staining of peritesticular adipose tissue sections (Scale bar: 100 μm). **(h)** adipocyte diameter size. **(i)** Oil red O staining of lipid droplets in liver (Scale bar: 100 μm). **(j)** Oil Red O percent stained area. Data were mean ± SEM, *n* = 5. Pound indicated significant differences between LFD vs. HFD (^#^*p* < 0.05; ^##^ *p* < 0.01; ^###^*p* < 0.001). Asterisk indicated significant differences between HFD vs. HFD+IPA (*p < 0.05; **p < 0.01; ***p < 0.001).

The weight gain of mice in the HFD group was significantly higher than that in the LFD group, and the administration of IPA significantly alleviated this trend (Figure 6a). There were no significant differences in food intake of mice among the three groups (Figure 6b). The mice in the HFD group had higher energy intake than that in the LFD group. No significant differences were observed in the energy intake of the mice between the HFD group and the HFD+IPA group (Figure 6c). IPA treatment was effective in reducing liver weight and liver index in mice (Figures 6d, e). The weight of peritesticular fat was significantly decreased by IPA intervention (Figure 6f). The diameter of adipocytes in the HFD group was increased compared with that in the LFD group, while IPA treatment significantly reduced the diameter of adipocytes (Figures 6g, h). Oil Red O staining revealed that IPA treatment effectively reduced hepatic lipid accumulation (Figures 6i, j). Consequently, these findings demonstrated that IPA prominently alleviated HFD-induced obesity and liver steatosis.

### 3.7 IPA ameliorated chronic inflammation and intestinal barrier damage in obese mice

**Figure 7:**
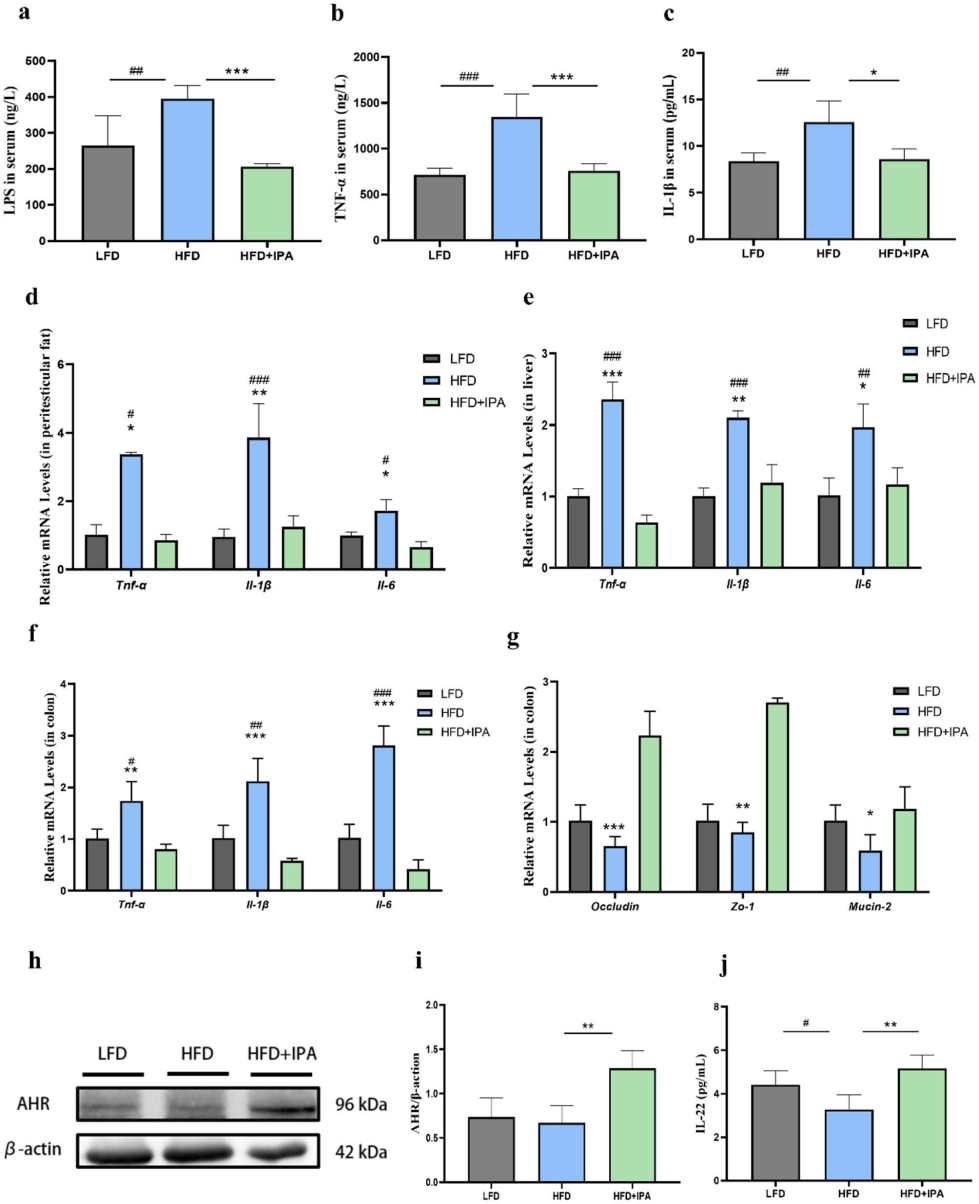
The effects of IPA on endotoxemia and intestinal barrier function in mice fed with high-fat diet. **(a)** LPS level in mice serum. **(b, c)** The levels of TNF-α and IL-1β in serum. **(d)** Expression levels of genes associated with inflammation in peritesticular adipose tissue. **(e)** Expression levels of genes associated with inflammation in liver. **(f)** Expression levels of genes associated with inflammation in colon. **(g)** Expression levels of intestinal barrier genes in colon. **(h, i)** AhR protein expression in colon. **(j)** IL-22 level in colon. Data were mean ± SEM, *n* = 5. Pound indicated significant differences between LFD vs. HFD (^#^*p* < 0.05; ^##^*p* < 0.01; ^###^*p* < 0.001). Asterisk indicated significant differences between HFD vs. HFD+IPA (*p < 0.05; **p < 0.01; ***p < 0.001).

HFD significantly increased the LPS, TNF-α, and IL-1β levels in serum, which were reversed upon IPA treatment (Figures 7a–c). The mRNA expressions of *Tnf-*α, *Il-6, Il-1*β were increased in liver, colon, and adipose tissue of mice in HFD group, and those elevation were reversed by IPA supplement (Figures 7d–f). Additionally, we found a significant decrease in mRNA expressions of *Occludin, Zo-1*, and *Mucin-2* in colon of mice in the HFD group, and their expression levels were up-regulated after IPA treatment (Figure 7g). Expectedly, IPA was able to correct the impaired AhR activity in obese mice (Figures 7h, i). It was also found that IPA supplementation could significantly increase the expression of IL-22 in colon, which was consistent with the results of quercetin supplementation (Figure 7j).

### 3.8 Quercetin improved gut barrier function by influencing IPA production

**Figure 8:**
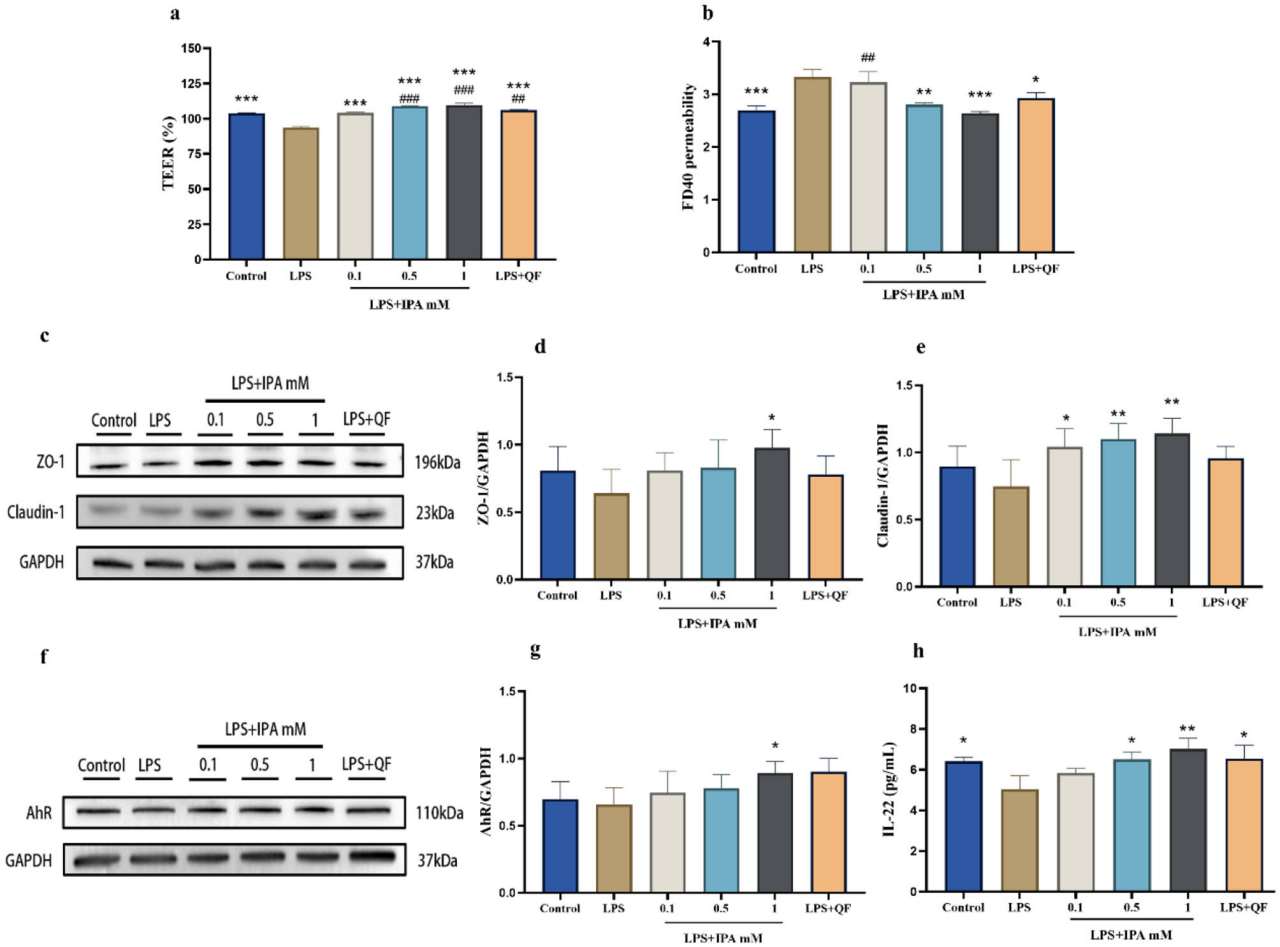
Quercetin improved gut barrier function through the IPA mediated-AhR activation in Caco-2 cells. Caco-2 cells were incubated for 24 h with complete DMEM medium from the following groups: control (no treatment), LPS, LPS+IPA (LPS co-cultured with varying concentrations of IPA), and LPS+QF (LPS combined with fecal diluent from quercetin-treated mice). **(a)** The TEER value. **(b)** The permeability of cell monolayers. **(c–e)** The protein expressions of ZO-1 and Claudin-1 in Caco-2 cells. **(f, g)** The protein expression of AhR in Caco-2 cells. **(h)** IL-22 level in Caco-2 cells. Data were mean ± SEM. Pound indicated significant differences vs. the control group (^##^*p* < 0.01; ^###^*p* < 0.001). The asterisk indicated significant differences vs. the LPS group (*p < 0.05; **p < 0.01; ***p < 0.001).

As mentioned above, our study found that quercetin significantly elevated the level of IPA in feces of mice. To determine whether quercetin enhances gut barrier function via AhR activation by the microbial tryptophan metabolite IPA, Caco-2 cell monolayers were incubated with IPA and fecal diluent from quercetin-treated mice (QF), alongside a control group without any drug treatment. After stimulation with LPS, TEER in Caco-2 cell monolayers was significantly decreased and paracellular permeability was increased. However, 1 mM IPA was able to significantly reverse this phenomenon. Interestingly, QF also dramatically increased TEER value and reduced paracellular permeability (Figures 8a, b). After LPS stimulation, a significant decrease in ZO-1 and claudin-1 protein expression was observed in Caco-2 cell monolayers. Treatment with IPA and QF can restore the expression of these proteins (Figures 8c–e). IPA can increase the production of IL-22 by activating AhR (24). Thus, we measured the expression of AhR and IL-22 in Caco-2 cells, and the results showed that AhR and IL-22 levels were decreased by LPS stimulation, while these changes were significantly antagonized by treatment with IPA and QF (Figures 8f–h). In summary, the above findings suggest that quercetin may improve gut barrier function by influencing the production of IPA, which leads to activation of the AhR/IL-22 pathway.

## 4 Discussion

Dietary intervention based on gut microbiota regulation has been recommended as a potential treatment for obesity (25). The growing interest in identifying beneficial gut microbiota metabolites and exploring their mechanisms for obesity intervention is driven by their critical roles in regulating glucose-lipid homeostasis, energy metabolism, and inflammatory responses (26). For instance, alterations in tryptophan metabolites have been linked to obesity. Previous studies have shown that gut microbiota-derived tryptophan metabolites are associated with obesity-related inflammation and gut barrier function (9). Tryptophan is metabolized by the gut microbiota into indole derivatives, which can modulate appetite and energy metabolism in the body (27). *In vitro* and *in vivo* studies demonstrate that indole derivatives of tryptophan metabolites enhance intestinal epithelial barrier function by upregulating genes critical to epithelial integrity (e.g., via pregnane X receptor and AhR signaling pathways), while also promoting goblet cell differentiation and mucus secretion to attenuate inflammatory responses in mice (28). Interestingly, quercetin, a widely used natural compound with antioxidant properties, ameliorates obesity, and associated inflammation by modulating the gut microbiota and metabolites to repair gut barrier damage (29), although the underlying mechanism remains incompletely understood. Thus, we hypothesized that quercetin mitigated HFD-induced obesity through modulation of microbial metabolite IPA, which further activated AhR/IL-22 pathway to improve intestinal barrier function.

Chronic low-grade inflammation is an important feature of obesity, involving the accumulation of macrophages in adipose tissue and the recruitment of additional inflammatory factors such as TNF-α and IL-1β (30). In addition, HFD can impair the barrier integrity of the intestinal mucosa, thereby facilitating the transfer of LPS into the systemic circulation (31). In this study, quercetin attenuated HFD-induced body weight gain, hepatic lipid deposition, intestinal barrier damage, and chronic inflammation, which was in line with our predicted results. It suggested that quercetin alleviated obesity by improving intestinal barrier function and reducing chronic low-grade inflammation.

Disturbed lipid metabolism in obesity may be associated with a reduced ability of the gut microbiota to convert tryptophan into AhR agonists (32). Defective activation of the AhR leads to decreased production of IL-22, which contributes to increased intestinal permeability and LPS translocation (33). Emerging evidence suggests that plant-derived quercetin has been shown to act as an AhR ligand to restore epithelial integrity by inducing the expression of tight junction proteins, thereby improving ulcerative colitis (34, 35). As shown in this study, quercetin intervention increased gene expression of tight junction proteins, as well as the expression of AhR and IL-22 in the colon. These results further suggested that quercetin could enhance the intestinal barrier function by activating AhR-dependent IL-22 expression.

The gut microbiota plays an important role in regulating microenvironmental homeostasis. HFD alters the composition and metabolic capacity of the gut microbiota, and reduces the quantities of microbial tryptophan metabolites. It is worth noting that AhR agonists derived from microbiota are mainly produced by *Peptostreptococcus russellii* and *Lactobacillus*, acting in the distal small intestine and colon (36). The results of our study were similar to previous reports, showing that quercetin treatment significantly enriched the abundance of *Faecalibacterium, Lactobacillus*, and *Dubosiella*. Up-regulated *Lactobacillus* strains increase AhR ligands and improve intestinal barrier function, leading to a reduction in obesity and metabolic disorders caused by high-fat diets (37). While supplementation with *Lactobacillus*, or its metabolite indole-3-lactic acid, can attenuate intestinal inflammation and restore IL-22 levels has been reported (21). Our data demonstrated that the dysregulated microbial tryptophan metabolism could be significantly restored by quercetin, which could be attributed to its regulatory role on gut microbiota. Moreover, IPA acts as a ligand of AhR, which is associated with anti-inflammatory effects (38). In this study, quercetin increased the abundance of *Lactobacillus*, which has been demonstrated to contribute to enhanced IPA production and AHR activation (39).

In this present study, we found that IPA could protect against HFD-induced obesity and hepatic steatosis. IPA induced the expression of tight junction proteins, leading to a reduction in levels of plasma endotoxin, and proinflammatory cytokines, which were consistent with previous research (40). Notably, recent epidemiological studies have shown that serum IPA levels are associated with obesity (9). Quercetin intervention increased IPA levels in serum and fecal dilution, which was consistent with the epidemiological investigation. Furthermore, IPA intervention elevated AhR and IL-22 protein expression in the colon. As evidenced by the numerous studies that IPA acts as an AhR ligand to increase IL-22 secretion (41), we hypothesized that quercetin improved obesity through influence in IPA production and activation of AhR/IL-22 axis.

We employed a Caco-2 cell monolayer model to confirm whether quercetin -mediated IPA was responsible for the improvement in the intestinal barrier function. IPA promoted the expression of tight junctions against the LPS-induced reduction observed in our study, confirming that IPA improves barrier integrity, and epithelial permeability in Caco-2 cells, consistent with the findings of Li et al. (42). Our data confirmed that QF enhanced gut barrier function and induced AhR expression, suggesting that the activation of AhR by the microbial metabolite IPA was mainly attributed to the regulatory effect of quercetin on gut microbiota. Consistent with previous studies, IPA and QF could improve intestinal barrier function by the activation of AhR to stimulate the expression of IL-22 in Caco-2 cells. Taken together, these results suggested that quercetin activated AhR/IL-22 pathway through microbiota-derived metabolite IPA, which plays an important role in improving gut barrier function.

Dietary polyphenols, such as quercetin, may alleviate obesity and metabolic disorders through gut microbial remodeling (16). Due to limited gastric and small intestinal absorption, most bound polyphenols are converted by gut microbiota into bioactive metabolites for systemic use, while only a small fraction of free polyphenols is directly metabolized (43, 44). However, our study highlights the unique role of quercetin in modulating the gut microbiota. Specifically, quercetin ameliorated high-fat diet-induced gut microbiota disruption by reversing the reduction in Lactobacillus abundance and IPA levels, ultimately protecting gut barrier function through activation of the AhR/IL-22 pathway and alleviating chronic inflammation and obesity. These results further demonstrate that the anti-obesity effects of quercetin are likely to be mediated by its regulation of the gut microbiota and metabolites, which is closely linked to the *in vivo* bioavailability and metabolic transformation of quercetin. The dose of quercetin and IPA used in this study (50 mg/kg/day) was chosen based on previous studies, supported by our pre-test observations showing no adverse effects (45, 46). Previous studies have shown that metabolomic changes can be reversed by combining different doses of quercetin and resveratrol in obesity models (47). Despite the findings of the present study demonstrating that quercetin and IPA (at a dose of 50 mg/kg/day) led to significant improvements in adiposity and inflammatory parameters, further investigation is required to ascertain the dose-response relationship between quercetin and IPA when administered as a standalone intervention in obese mice. Moreover, this study also lacked AhR-knockout mice models and fecal microbiota transplantation experiments, which could provide further insight into the underlying mechanisms. Future exploration of the regulatory mechanisms of quercetin on microbial tryptophan metabolism should be continued.

## 5 Conclusions

In conclusion, quercetin supplementation restores high-fat diet induced gut microbiota dysbiosis, subsequently increasing IPA level to activate the AhR/IL-22 signaling pathway, thereby enhancing intestinal barrier integrity and attenuating chronic inflammation, which collectively ameliorate obesity. This research highlights the potential of quercetin as a dietary supplement for the prevention and treatment of obesity.

## Data Availability

The datasets presented in this study can be found in online repositories. The names of the repository/repositories and accession number(s) can be found in the article/supplementary material.
